# Population Disequilibrium as Promoter of Adaptive Explorations in Hepatitis C Virus

**DOI:** 10.3390/v13040616

**Published:** 2021-04-03

**Authors:** Carlos García-Crespo, Isabel Gallego, María Eugenia Soria, Ana Isabel de Ávila, Brenda Martínez-González, Lucía Vázquez-Sirvent, Rebeca Lobo-Vega, Elena Moreno, Jordi Gómez, Carlos Briones, Josep Gregori, Josep Quer, Esteban Domingo, Celia Perales

**Affiliations:** 1 Centro de Biología Molecular “Severo Ochoa” (CBMSO) (CSIC-UAM), Consejo Superior de Investigaciones Científicas (CSIC), Campus de Cantoblanco, 28049 Madrid, Spain; carlos.garciac@cbm.csic.es (C.G.-C.); igallego@cbm.csic.es (I.G.); mariae.soriab@quironsalud.es (M.E.S.); aideavila@cbm.csic.es (A.I.d.Á.); elena.moreno86@gmail.com (E.M.); 2 Centro de Investigación Biomédica en Red de Enfermedades Hepáticas y Digestivas (CIBERehd) del Instituto de Salud Carlos III, 28029 Madrid, Spain; jgomez@ipb.csic.es (J.G.); cbriones@cab.inta-csic.es (C.B.); josep.gregori@gmail.com (J.G.); josep.quer@vhir.org (J.Q.); 3 Department of Clinical Microbiology, Instituto de Investigación Sanitaria-Fundación Jiménez Díaz University Hospital, Universidad Autónoma de Madrid (IIS-FJD, UAM), Av. Reyes Católicos 2, 28040 Madrid, Spain; brenda.martinez@quironsalud.es (B.M.-G.); lucia.vazquez@quironsalud.es (L.V.-S.); rebeca.lobo@quironsalud.es (R.L.-V.); 4 Instituto de Parasitología y Biomedicina “López-Neyra” (IPBLN, CSIC), Parque Tecnológico Ciencias de la Salud, Armilla, 18016 Granada, Spain; 5 Centro de Astrobiología (CAB, CSIC-INTA), Torrejón de Ardoz, 28850 Madrid, Spain; 6 Liver Unit, Internal Medicine Hospital Universitari Vall d’Hebron, Vall d’Hebron Institut de Recerca (VHIR), 08035 Barcelona, Spain; 7 Roche Diagnostics, S.L., Sant Cugat del Vallés, 08174 Barcelona, Spain

**Keywords:** viral quasispecies, hepatitis C virus, mutational waves, residue conservation, sequence space, antiviral drug resistance, universal vaccines, COVID-19

## Abstract

Replication of RNA viruses is characterized by exploration of sequence space which facilitates their adaptation to changing environments. It is generally accepted that such exploration takes place mainly in response to positive selection, and that further diversification is boosted by modifications of virus population size, particularly bottleneck events. Our recent results with hepatitis C virus (HCV) have shown that the expansion in sequence space of a viral clone continues despite prolonged replication in a stable cell culture environment. Diagnosis of the expansion was based on the quantification of diversity indices, the occurrence of intra-population mutational waves (variations in mutant frequencies), and greater individual residue variations in mutant spectra than those anticipated from sequence alignments in data banks. In the present report, we review our previous results, and show additionally that mutational waves in amplicons from the NS5A-NS5B-coding region are equally prominent during HCV passage in the absence or presence of the mutagenic nucleotide analogues favipiravir or ribavirin. In addition, by extending our previous analysis to amplicons of the NS3- and NS5A-coding region, we provide further evidence of the incongruence between amino acid conservation scores in mutant spectra from infected patients and in the Los Alamos National Laboratory HCV data banks. We hypothesize that these observations have as a common origin a permanent state of HCV population disequilibrium even upon extensive viral replication in the absence of external selective constraints or changes in population size. Such a persistent disequilibrium—revealed by the changing composition of the mutant spectrum—may facilitate finding alternative mutational pathways for HCV antiviral resistance. The possible significance of our model for other genetically variable viruses is discussed.

## 1. Introduction: HCV Quasispecies in Cell Culture and In Vivo

RNA viruses replicate as complex mutant spectra termed viral quasispecies. This designation is based on the close similarity of the population structure and dynamics of present day riboviruses and retroviruses, and those of primitive replicons at the onset of life, as postulated by quasispecies theory [1,2,3]. A quasispecies attribute of biological relevance for viruses is that, at any given time point, a viral population contains multitudes of genotypic and phenotypic variants, present at different relative frequencies. The coexisting variant multitudes have been indistinctly termed mutant distributions, spectra, swarms or clouds. From analyses of sequential viral isolates replicating in cell culture or in host organisms, the variant composition is highly dynamic. This introduces an indetermination regarding the precise composition of viral populations. Genomes present at low frequency in a mutant spectrum may influence the behavior of the population ensemble, and sometimes emerge to dominance through positive selection or bottleneck events. The latter are often associated with host-to-host transmission, and contribute to virus diversification. In this manner, the indetermination inherent to mutant swarms is projected into virus epidemiology. The information provided by the consensus sequence of a population (in which the underlying mutant cloud is not represented) is fragmentary regarding the understanding of virus–host interactions. Indeed, the same consensus sequence may correspond to different mutant genome repertoires [4].

Despite the relevance of mutant clouds, the number of different mutations, their frequency, and their distribution among genome classes in evolving viruses are largely unknown parameters, partly due to technical challenges. Only recently, the application of ultra-deep sequencing (UDS) methodologies has allowed some (although still limited) penetration into the composition of viral mutant spectra [5,6,7,8]. In our laboratories, we have approached population dynamics of HCV with the aim of increasing our understanding of quasispecies implications through determination of diversity indices in a comparative manner in cell culture and in vivo [9,10,11,12,13,14,15,16]. In cell culture, the main question we asked is how the HCV mutant spectrum composition would change upon prolonged replication in the relatively simple and stable biological environment provided by an established human hepatoma cell line, in the absence of external perturbations. Prolonged replication meant 200 serial passages, which are equivalent to about 700 days of continuous HCV replication. To our knowledge, the effects of such extensive replication in the absence of external perturbations have not been investigated with any other viral pathogen. In our serial infection design, HCV was the only evolving entity since evolution of infected cells was prevented, although a certain degree of cellular heterogeneity was unavoidable [12,17]. A counterpart was provided by genomic sequences of HCV replicating in infected patients, where the virus confronts complex and variable environments. These studies have evidenced both common and distinct features of HCV quasispecies in cell culture and in vivo; for example, while mutant spectra were of comparable average complexity, residues that tend to be highly variable in vivo—i.e., the hypervariable regions 1 and 2, as defined on the basis of sequence comparisons of clinical samples—showed little variation upon prolonged replication in Huh-7.5 cells [12,13,14,15].

The following three observations drawn from our previous work (Table 1) merit being emphasized: (i) Mutant spectrum complexity in cell culture remained high, independently of the degree of adaptation of the virus to the cellular environment; the adaptation was quantified by comparing the replicative fitness of HCV from early and late passages [12,14,17,18]. The expectation that long-term replication in the same environment would prune away ill-adapted genomes to perpetuate a limited, high fitness subset—thus narrowing the mutant cloud—was not fulfilled. Moreover, the frequency of individual genomic mutations exhibited changes that did not subside—if anything they became more prominent—when the virus increased its adaptation to the cell culture environment. We referred to these modifications in mutant frequency as mutational waves that concerned 145 different mutations that mapped within the 1005 residues analyzed from the NS5A-NS5B-coding region; the term emphasizes that variations in mutant frequency were permanent along the 200 passages [12,14].

(ii) The second noteworthy observation was that the great majority of residues examined (both at the nucleotide or deduced amino acid level) that ranked as conserved in the HCV sequence alignments of the Los Alamos National Laboratory (LANL) data base were variable in mutant spectra from cell culture or from infected patients. Details on how this comparison were performed have been previously published [16], and are also described in Materials and Methods (Section 2). Residue conservation defined according to sequence alignments in data banks is often elevated to the rank of a general attribute of a given residue, genomic region or protein domain. Conservation is equated with functional essentiality and, consequently, the relevant sites are considered the best antiviral targets because their variations would be incompatible with the survival of escape mutants. Little attention has been put into the possibility that residue conservation in mutant spectra from infected patients differs from the degree of conservation deduced from data bank alignments. However, mutant spectra of any RNA virus are the real biological entities towards which a vaccine should protect and antiviral agents act to suppress the viability of the ensemble. For this reason, we recently examined this question with HCV. We found that there is no agreement between amino acid conservation according to the alignments of sequences present in mutant spectra from infected patients and alignments in the LANL data bank. The result was the same whether the sequence comparison considered multiple HCV genotypes or only individual genotypes [16]. The term “conservation” applied to residues of RNA viral genomes has a limited meaning since it depends not only on the evolutionary history of the virus but also on the sequence resolution level applied (mutant spectra, consensus sequences or sequence alignments in data banks). From a practical stand-point, the discordance raises considerable uncertainty about the expected success of antiviral universal vaccines and pan-genotypic antiviral agents. These are rising fields in clinical virology and vaccinology [20,21,22,23,24,25,26,27,28,29]. We argue that such designs should take into consideration a new criterion of conservation based on the alignment of sequences present in mutant spectra [16].

(iii) A third observation, which falls into the domain of patient’s clinical management, is that we identified a number of highly represented substitutions (HRS) at the amino acid level associated with HCV treatment failures that did not correspond to the standard resistance-associated substitutions (RAS). In contrast to RAS, HRS tend to be conservative according to replacement acceptability matrices, and they may facilitate HCV resistance to different direct acting antiviral (DAA)-based treatments. The presence of HRSs suggests the operation of escape mechanisms different from the most common RAS selection [15].

The present article reviews the previous evidence that supports that the three observations summarized above have a common origin in viral population dynamics, as predicted by quasispecies theory. We also present new data that document the occurrence of mutational waves in HCV populations passaged in cell culture in the absence of drugs and in the presence of sub-inhibitory concentrations of the antiviral agents favipiravir or ribavirin. Mutational waves were observed under all passage conditions. Second, concerning the HCV mutant repertoires in infected patients, we show by ultra-deep sequencing (UDS) of NS3-NS5A amplicons that amino acid conservation in mutant spectra does not fit the conservation pattern indicated by the LANL data base. These results reinforce our previous results with NS5A-NS5B amplicons with the same patient cohort [16]. We describe a new calculation that shows that amino acids that rank as conserved in mutant spectra from infected patients correspond to high conservation groups in the LANL alignment. This agreement opens a possibility of implementation of new amino acid sequence alignments that highlight “super-conserved” residues for variable viral pathogens, that take into account both standard consensus sequences stored in data banks and the available mutant spectra. With the extended evidence reported here, we hypothesize that the three observations summarized above (occurrence of mutational waves, incongruences of residue conservation scores, and the presence of HRS in patients who failed therapy) have a common origin in one of the predictions of quasispecies theory.

## 2. Materials and Methods

### 2.1. Origin of the Hepatitis C Virus Populations, and Infection of Huh-7.5 Cells

The initial HCVcc population for studies on experimental evolution in cell culture was obtained by transcription of plasmid Jc1FLAG2(p7-nsGluc2A) [30], followed by RNA electroporation into Huh-Lunet cells, concentration of the progeny virus shed into the culture medium, and further amplification in Huh-7.5 reporter cell monolayers. The latter step yielded the parental population HCV p0 [17] that has been used in subsequent studies, including those reported here. HCV p0 was passaged up to 200 times in Huh-7.5 reporter cells, as previously described [12]. HCV infectivities were calculated with the 50% tissue culture infective dose 50 (TCID_50_) [31]. Briefly, in each passage, 4 × 10^5^ fresh cells were infected by the virus in the cell culture medium of the previous infection; the multiplicity of infection (MOI) ranged from 0.1 to 0.5 TCID_50_ per cell. New series of parallel passages were initiated from HCV p0, HCV p100, and HCV p200, in the absence of any drug or in the presence of either favipiravir (400 µM) or ribavirin (100 µM).

**Figure 1:**
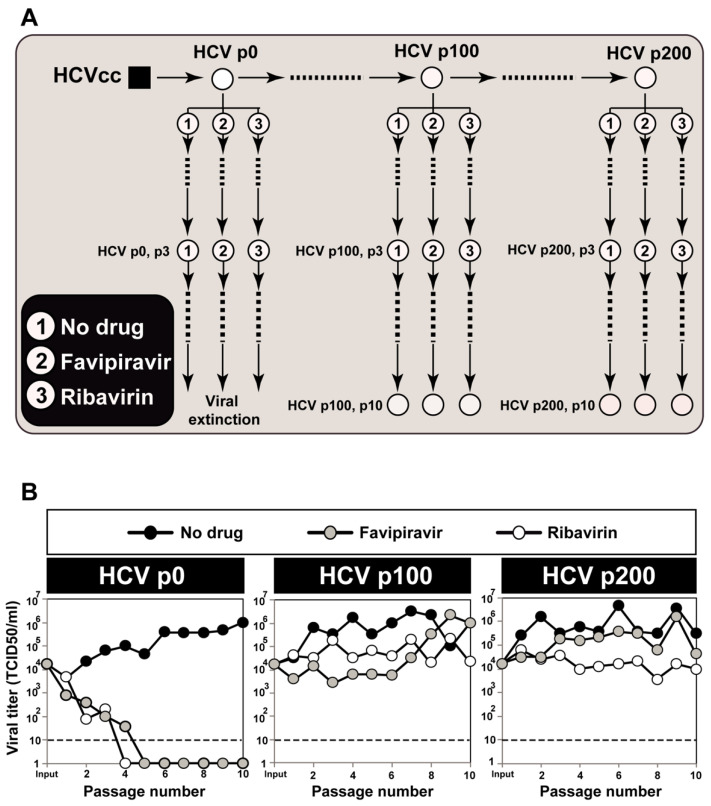
Passage of hepatitis C virus in the absence or presence of favipiravir or ribavirin. (**A**) scheme of the serial passages starting from populations HCV p0, HCV p100, and HCV p200. The origin of each virus is described in the text. Numbers inside circles identify the drug present in each viral lineage (inserted box). Each population depicted by an encircled number was analyzed by UDS to quantify mutations and draw mutational waves. For HCV p0, the analyses were restricted to the initial populations because, at later passages, favipiravir and ribavirin led to virus extinction [19,34]; (**B**) viral titers in the Huh-7.5 reporter cell culture supernatants quantified for the populations depicted in (**A**). These infectivity data have been previously published [19], and are included here for completeness.

To initiate the serial passages, 4 × 10^5^ Huh-7.5 reporter cells were pretreated with the nucleoside analogue (or Dulbecco’s modification of Eagle’s medium for infections in absence of drugs) for 16 h prior to infection at a MOI of 0.03 TCID_50_ per cell; the time of virus adsorption to the cells was 5 h. For subsequent passages, 4 × 10^5^ Huh-7.5 reporter cells were pretreated with the drugs as for the initial infection, and infected with the virus contained in 0.5 mL of the cell culture medium from the previous infection of the same lineage; viral titers indicated that the MOI ranged from 4.6 × 10^−5^ to 6 TCID_50_ per cell (the MOI varied depending on the virus and the effect of the inhibitors, and the value for each infection can be calculated from the titrations reported in the experiments). In all passages, infections were allowed to proceed for 72 h to 96 h. Additional procedures, including titration of infectivity to determine TCID_50_ values, viral RNA quantification, and HCV extinction criteria, have been previously described [32,33,34]. The passage scheme and virus titers are shown in Figure 1.

### 2.2. RNA Extraction, Viral RNA Amplification, and Ultra-Deep Sequencing of Cell Culture Populations

**Figure 2:**
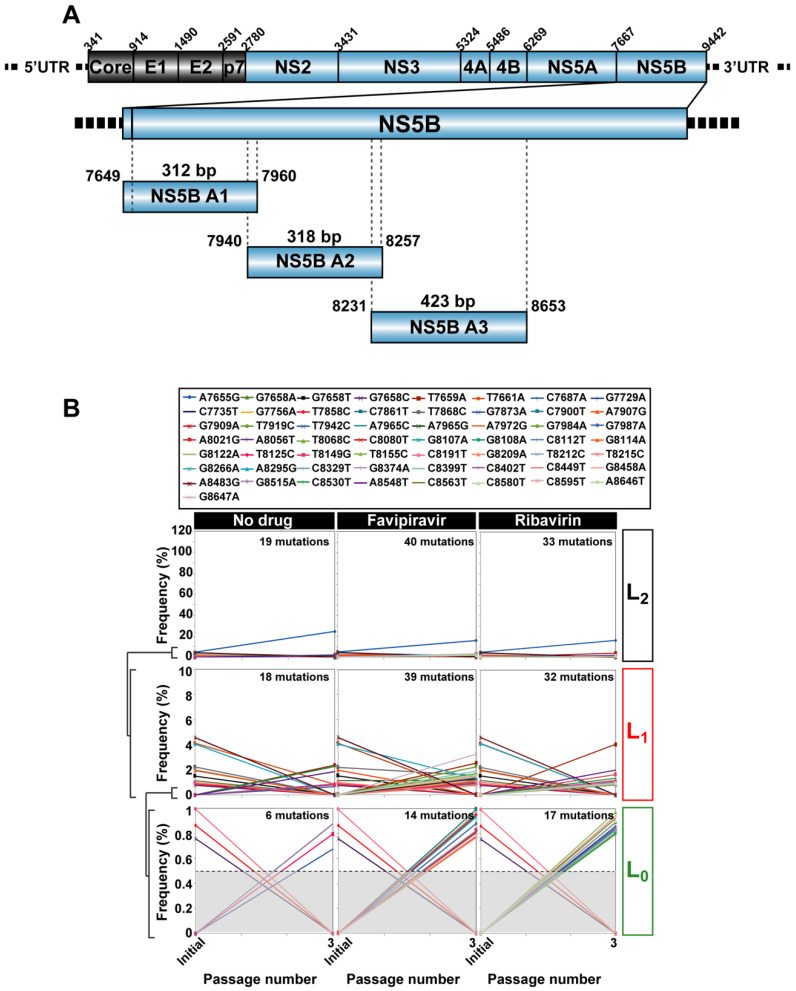

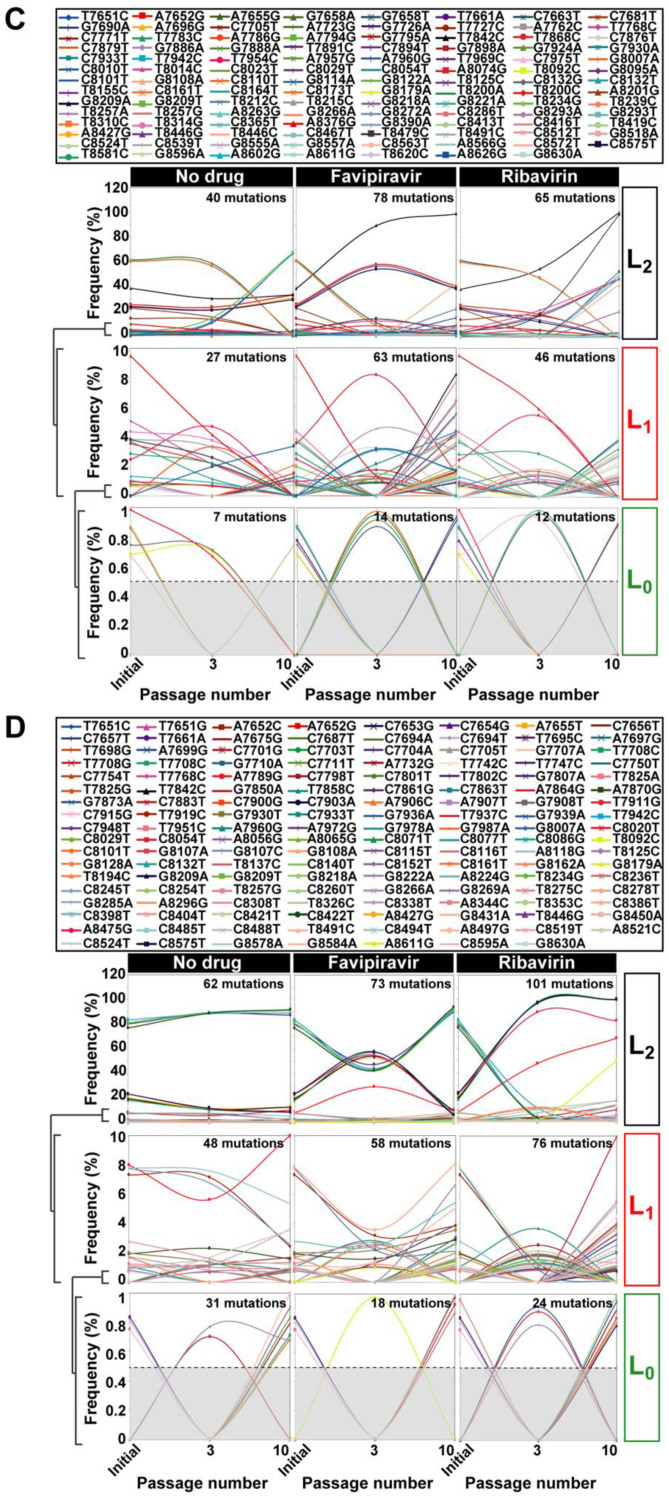
Mutational waves in hepatitis C virus in the presence of antiviral inhibitors. (**A**) scheme of the HCV genome, encoded proteins, and location of the amplicons used for UDS analysis; residue numbering is according to reference isolate JFH-1; (**B**) top box: mutations (color coded) that changed in frequency upon serial passage of HCV p0. The panels below show the quantification of mutant frequencies in the absence or presence of the antiviral drug, indicated at the top. The range of frequency variations has been divided into levels L_0_, L_1_, and L_2_, as shown in ordinate, and explained in the text; passage number is given in abscissa, and the total number of mutations at each level and passage condition is written inside the panel; the discontinuous horizontal line in the L_0_ panels indicates the 0.5% cut-off frequency value used in these experiments; (**C**) same as (**B**) except that the populations analyzed were those derived from HCV p100; (**D**) same as (**B**) except that the populations analyzed were those from HCV p200; note the line bundles particularly visible at level L_2_. The same mutant frequency scales in ordinate are used for each virus and level, for comparative purposes. The complete list of mutations and deduced amino acid substitutions is given in Tables S3–S5 of [19]. The experimental design is depicted in Figure 1, and experimental and bioinformatics procedures are described in the text.

Intracellular viral RNA was extracted from the initial and passaged populations (Qiagen RNeasy kit, Qiagen, Valencia, CA, USA), and amplified by RT-PCR using Accuscript (Agilent) and specific HCV oligonucleotide primers (described in Table S10 of [14]). Amplification products were analyzed by agarose gel electrophoresis, using Gene Ruler 1 Kb Plus DNA ladder (Thermo Scientific, Waltham, MA, USA) as molar mass standard. Negative controls without template RNA were included to ascertain the absence of contaminating templates. To avoid complexity biases due to redundant amplifications of the same initial RNA templates ensuring an excess of template in the RT-PCR reactions, amplifications were carried out with template preparations diluted 1:10, 1:100 and 1:1000; only when at least the 1:100 diluted template produced a visible DNA band was molecular cloning pursued using the DNA amplified from undiluted template. PCR products were purified (QIAquick Gel Extraction Kit, QIAgen), quantified (Pico Green assay), and tested for quality (Bioanalyzer DNA 1000, Agilent Technologies, Santa Clara, CA, USA) prior to Illumina UDS analysis (MiSeq platform, with the 2 × 300-bp mode with v3 chemistry). The amplicons analyzed span HCV genomic nucleotides 7649 to 8653 (residue numbering according to reference isolate JFH-1; accession number #AB047639) that correspond to amino acid 461 of NS5A to amino acid 329 of NS5B (Figure 2A).

### 2.3. RNA Extraction, Viral RNA Amplification, and Ultra-Deep Sequencing of HCV from Infected Patients

Viral RNA was extracted from 140 µL of patient plasma/serum samples (Qiagen QIAamp Viral RNA Mini Kit, Qiagen, NV, Venlo, The Netherlands) or automatically using a total nucleic acid isolation Kit in a COBAS/AmpliPrep system (Hoffman-La Roche Ltd., Basel, Switzerland) from a cohort of 220 patients described in [13,15], and amplified by RT-PCR using Transcriptor One Step RT-PCR kit (Roche Applied Science) followed by an internal PCR using FastStart Taq DNA polymerase (Roche Applied Science) with specific HCV oligonucleotide primers covering the three regions of interest (NS3, NS5A and NS5B) (described in Tables S4–S6 of [11]). Amplification products were analyzed by agarose gel electrophoresis, using Gene Ruler 1 Kb Plus DNA ladder (Thermo Scientific) as molar mass standard. Negative controls without template RNA were included to ascertain the absence of contaminating templates. PCR products were purified (QIAquick Gel Extraction Kit, QIAgen), quantified (Qubit TM dsDNA Assay Kit, Thermofisher Scientific), and tested for quality (Bioanalyzer DNA 1000, Agilent Technologies) prior to Illumina UDS analysis (MiSeq platform, with the 2 × 300-bp mode with v3 chemistry). The amplicons analyzed span HCV genomic nucleotides 3513 to 3956 for NS3, 6327 to 6713 for NS5A and 7971 to 8561 for NS5B (residue numbering according to reference isolate H77; accession number #AF009606) that correspond to amino acids 32 to 179 for NS3, 24 to 152 for NS5A, 124 to 320 for NS5B. For further details about ultra-deep sequencing procedures see [11].

### 2.4. Controls in the Ultra-Deep Sequencing Analyses

We have determined the following controls: (1) Determination of the basal error of the process that leads to mutant spectrum characterization. A known infectious clone was used to perform RT-PCR and the internal PCR and ultra-deep sequenced using MiSeq; (2) Determination of the frequency of PCR recombination in the course of the amplification steps using mixtures of wt and mutant DNA clones in known proportions, to perform RT-PCR and the internal PCR and ultra-deep sequenced using MiSeq; (3) Determination for similarity in read composition in different RT-PCR amplifications and sequencing runs. For HCV p0, HCV p100 and HCV p200 populations, and the populations passaged in the absence or presence of favipiravir and ribavirin, mutations identified with a frequency above the 0.5% cut-off value were considered for the analyses. For the case of patient samples, we established a conservative limit of detection for individual amino acid substitutions at 1% frequency. For additional details of the controls, see [10,11,35].

### 2.5. Amino Acid Conservation in Infected Patients versus Los Alamos Database (LANL)

Sequences from LANL were retrieved with the following the inclusion criteria: sequences that had been confirmed, that corresponded to full-length (or near-full length) genomes (without large insertions or deletions), and with no evidence of their being recombinants. Their HCV genotype/subtype distribution is: 553 sequences of genotype G1a; 427 of G1b; 3 of G1c; 33 of G2a; 81 of G2b; 8 of G2c; 5 of G2j; 4 of G2k; 49 of G3a; 17 of G4a; 5 of G4d; and 6 of G4f. Examination of the conservation category of the 148 amino acids of NS3 and 129 amino acids of the NS5A was performed using LANL. For the calculation of the conservation range of individual residues, no distinction has been made between HCV subtypes. HCV samples from a cohort of 220 patients assembled from 39 Spanish hospitals were analyzed by deep-sequencing [13]. The patients had failed DAA-based, interferon (IFN)-α free therapy. A comparison of amino acid conservation pattern deduced from the LANL alignment with the conservation observed in mutant spectra evolving in the cohort of infected patients was performed as in [16].

### 2.6. Statistics

The statistical significance of differences in mutations that participated in molecular waves, differences in mutations that followed the same trajectory in molecular waves and number of mutations that are unique or are shared by several HCV populations in the different drug conditions was calculated with the proportion test using software R version 3.6.2.

## 3. HCV Mutational Waves in the Presence of Mutagenic Nucleotide Analogues

There is clinic and laboratory evidence that ribavirin is mutagenic for HCV [32,36,37], and that favipiravir is mutagenic for several RNA viruses [38,39,40,41], including HCV [33]. We have previously documented that high fitness HCV (populations HCV p100 and HCV p200) exhibited decreased sensitivity to favipiravir and ribavirin, as compared with the initial, low fitness population HCV p0 [19]. Mutational waves were observed upon passage of the three HCV populations in the absence or presence of favipiravir (400 µM) or ribavirin (100 µM) (Figure 2B–D).

Mutation waves were divided into three nested levels according to the range of mutation frequencies involved: L_0_ (basal level, with frequencies up to 1%, L_1_ (frequencies up to 10%), and L_2_ (any frequency level) as was previously defined [14,42]. For HCV p0, only two points of analysis are available (initial population, and passage 3) because, at later passages, in the presence of either favipiravir or ribavirin, virus infectivity was very low, and eventually lost by lethal mutagenesis [19,32]. HCV p100 and HCV p200 could be subjected to ten passages in the presence of the analogues because the higher fitness of these populations relative to HCV p0 confers them resistance to antiviral inhibitors, including lethal mutagens [18,19,43] (see Section 4).

For HCV p0, the number of mutations that participated in molecular waves was significantly larger for most populations passaged in the presence of an inhibitor than in its absence (*p* = 0.0001 and *p* = 0.0054 for favipiravir and ribavirin, respectively; proportion test). The same comparison for HCV p100 yielded *p* = 7.287 × 10^−7^ and *p* = 0.0005, respectively, and for HCV p200, *p* = 0.1118 and *p* = 1.281 × 10^−5^, respectively (proportion test).

Some difference was observed between HCV p100 and HCV p200 regarding linkage of mutations that followed the same trajectory (visualized as mutation bundles in Figure 2). In the absence of drugs, taking the complete set of data assembled in level L_2_, the number of different mutations that followed the same trajectory was 61 (98.4% of the total wave mutations) for HCV p200, and 38 (95.0% of the total) for HCV p100, but the tendency did not reach statistical significance (*p* = 0.3489; proportion test). In the presence of favipiravir, the number of mutations with the same trajectory was 71 (97.3% of the total) for HCV p200, and 75 (96.2% of the total) for HCV p100 (*p* = 0.5; proportion test). In the presence of ribavirin, the number of mutations with the same trajectory was 98 (97% of the total) for HCV p200, and 59 (90.8% of the total) for HCV p100 (*p* = 0.08264; proportion test). Therefore, the differences did not reach statistical significance.

The interpretation of mutation frequency bundles requires further study, but they do not necessarily mean that mutations are linked in the same genome. This is an appealing possibility since it may denote a tendency towards some population equilibrium that is not (and probably cannot be) fully attained. However, substantiation of this possibility would require whole genome UDS with resolution and reliability similar to those attained with short amplicons, an objective that is still technically challenging.

**Figure 3:**
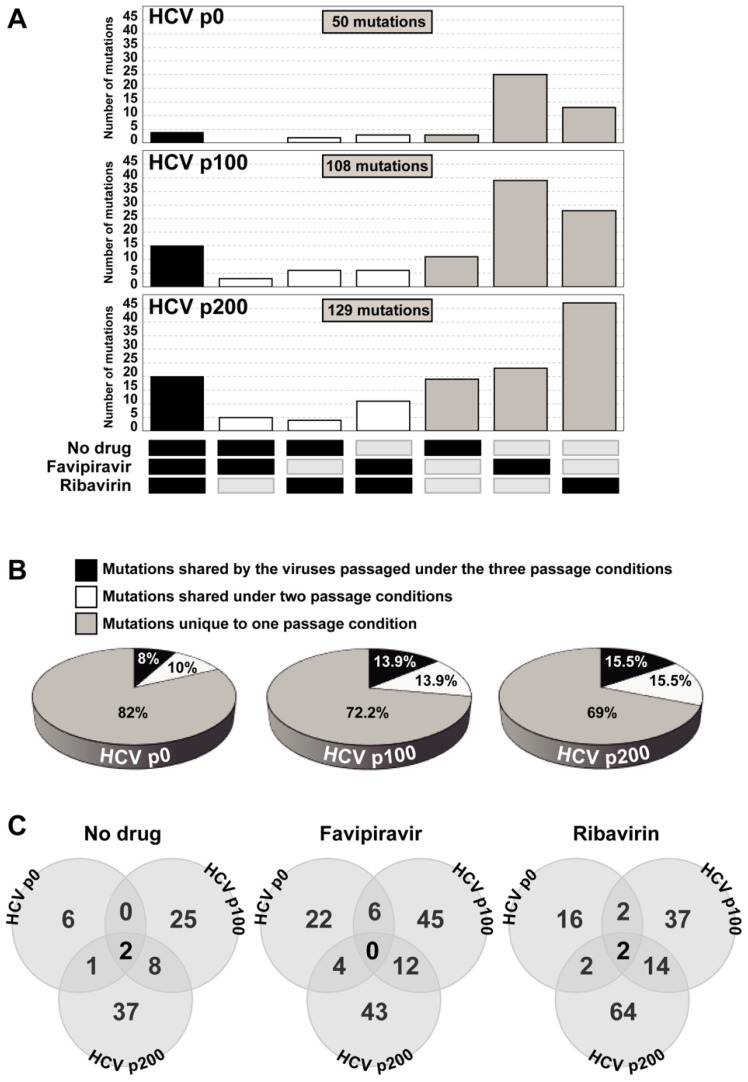
Shared and unique mutations in the hepatitis C virus populations passaged in absence or presence of inhibitors. (**A**) number of mutations (ordinate) that participated in mutational waves in populations derived from HCV p0, HCV p100, and HCV p200 (excluding mutations that appear only in the initial populations), which were shared among conditions of the same virus and also among viruses passaged in the absence or presence of inhibitors; the passage conditions in which mutations were found is indicated by the filled rectangles drawn below the abscissa; note that the majority of mutations was unique to one condition (grey bars). (**B**) summary of the percentage of unique and shared mutations in the populations derived from HCV p0, HCV p100, and HCV p200. The complete list of mutations and deduced amino acid substitutions is given in Tables S3–S5 of [19]. (**C**) Venn diagram of mutations that are shared among HCV p0, HCV p100 and HCV p200 in the three experimental conditions (no drug, favipiravir, and ribavirin). The experimental procedures are described in the text.

Evidence of instability of putative sub-lineages within mutant spectra is provided by the overwhelming number of mutations that are present only in HCV p0, HCV p100, or HCV p200 (and their derived populations passaged in absence or presence of mutagenic analogues) versus those shared by two or three of these population groups. The difference between the number of mutations that was present in only one of the virus populations versus the number of mutations that was shared by two or three populations was determined by a proportion test (see Section 2.6 in Materials and Methods) and yielded the following results. In the populations passaged in the absence of any drug: for HCV p0, *p* = 0.1729; for HCV p100, *p* = 0.000409; and for HCV p200 *p* = 1.671 × 10^−7^. In the populations passaged in in the presence of favipiravir: for HCV p0, *p* = 0.00298; for HCV p100, *p* = 1.806 ×10^−6^; and for HCV p200, *p* = 8.465 × 10^−7^. In the populations passaged in the presence of ribavirin: for HCV p0, *p* = 0.003328; for HCV p100, *p* = 0.0002991; and for HCV p200, *p* = 1.04 × 10^−12^ (Figure 3). We conclude that mutational waves are a feature of HCV evolution in the hepatoma cell culture22—with some accentuation in the presence of either of the two mutagenic agents tested—that persist until late passages, but with some suggestion of increased intra-population order in HCV p200 relative to HCV p100.

## 4. Amino Acid Conservation in HCV Quasispecies from Infected Patients

**Figure 4:**
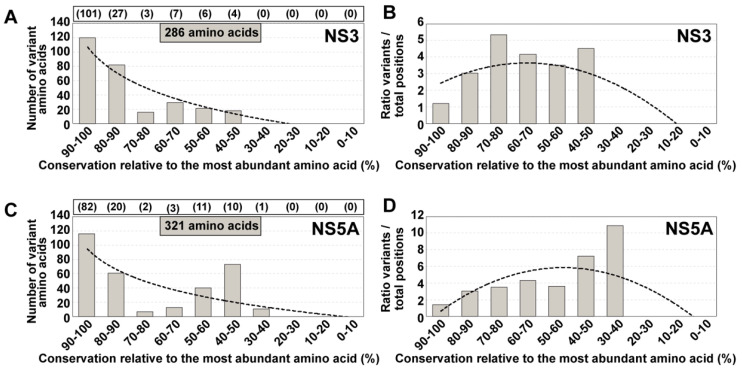
Distribution of positions with variant amino acids identified in HCV from infected patients among amino acid conservation groups according to the LANL amino acid sequence alignment. (**A**) Assignment for the 286 variant amino acids identified within the 148 amino acid stretch comprised between amino acids 32 to 179 of NS3. Conservation groups are indicated in abscissa, and the number of variants in each group is indicated in ordinate. The total number of amino acids that fall in each conservation category in the LANL alignment is indicated in parentheses in the upper box. The discontinuous line corresponds to function y = −51.97 ln(x) + 107.1 (R^2^ = 0.8821). (**B**) Same as (**A**), but with values normalized to the number of residues in each conservation group. The defining function is y = −0.1385x^2^ + 1.0988x + 1.4585 (R^2^ = 0.6001). (**C**,**D**) same as (**A**,**B**) but for 321 variant amino acids identified within the 129 amino acid stretch comprised between amino acids 24 to 152 of NS5A. The defining functions are: (**C**): y = −41.41ln(x) + 94.654 (R^2^ = 0.5865); (**D**): y = −0.2979x^2^ + 3.1082x − 2.2008 (R^2^ = 0.4371). The complete list of deduced amino acid substitutions recorded in the HCV-infected patients is given in Table S1 of [15].

The conclusion that the degree of amino acid conservation in HCV mutant spectra of infected patients did not fit the conservation status of the same residues in the LANL alignment was reached with UDS analysis of NS5B amplicons [16]. In the present study, we have extended the analysis to amino acids encoded by amplicons of the NS3- and NS5A-coding regions, using the same cohort of 220 HCV-infected patients [13]. The residues under study span amino acids 32 to 179 of protein NS3 (which correspond to genomic nucleotides 3513 to 3956; numbering according to HCV isolate H77), and amino acids 24 to 152 of protein NS5A (which correspond to genomic nucleotides 6327 to 6713; numbering according to HCV isolate JFH-1). The distribution of variant amino acids among conservation groups defined according the LANL alignment (Figure 4) confirms our previous conclusion with NS5B that the majority of amino acids that are variable in the mutant spectra of infected patients fall within the highest (80–90% and 90–100%) conservation ranges according to the LANL data bank. The only noticeable difference was the relative higher residue representation in the 40–50% conservation range for NS5A, not seen with NS3 and to a lesser extent with NS5B [16]. Therefore, we reaffirm the conclusion that conservation in data banks is not a reliable guide to predict conservation of amino acids in the HCV proteins of infected patients.

**Figure 5:**
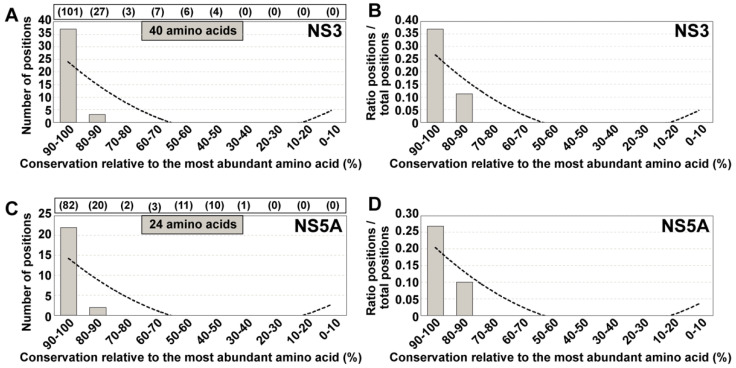
Distribution of conserved positions—identified by aligning the HCV mutant spectra from infected patients—among the amino acid conservation groups according to the LANL amino acid sequence alignment. (**A**) Assignment of the 40 strictly conserved amino acids patients’ quasispecies to conservation groups calculated from the LANL alignment. The residues under study span amino acid 32 to amino acid 179 of protein NS3. Conservation groups are indicated in abscissa, and the number of positions that fall into each group is indicated in ordinate. The total number of amino acids that fall in each conservation category in the LANL alignment is indicated in parentheses in the upper box. The discontinuous line corresponds to y = 0.8636x^2^ − 11.645x + 34.8 (R^2^ = 0.6351). (**B**) same as (**A**), but with values normalized to the total number of amino acids that fall within each conservation group. The defining function is y = 0.0092x^2^ − 0.1255x + 0.3853 (R^2^ = 0.7652); (**C**,**D**) same as (**A**,**B**) but with the 24 strictly conserved amino acids comprised between amino acid 24 and 152 of protein NS5A. The defining functions are: (**C**): y = 0.5152x^2^ − 6.9515x + 20.8 (R^2^ = 0.642); (**D**): y = 0.0069x^2^ − 0.0943x + 0.2915 (R^2^ = 0.7923). The complete list of those amino acids that are strictly conserved in the HCV-infected patients is given in Table S1.

**Figure 6:**
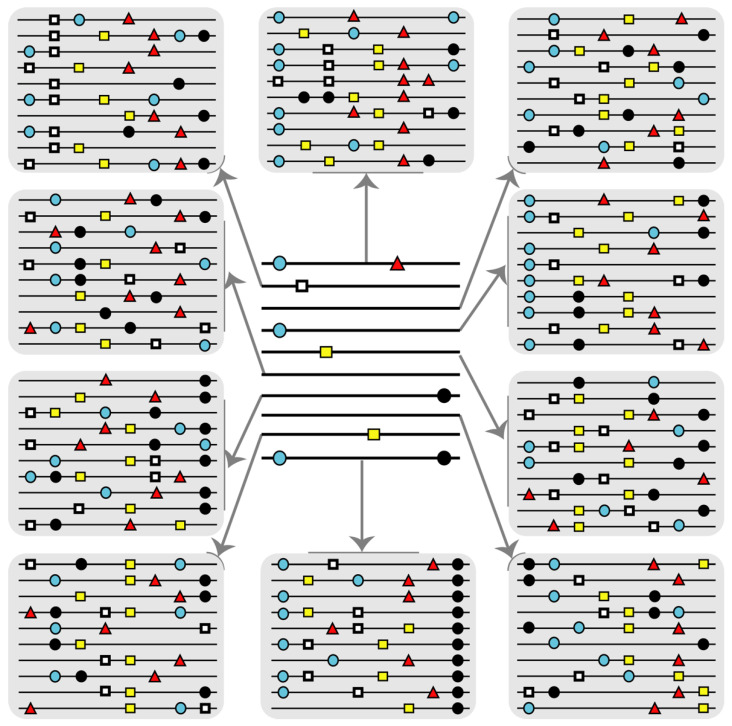
Scheme that illustrates the interest of incorporating information retrieved from mutant spectra into data bases for viruses. Each horizontal line represents a nucleotide or amino acid sequence with mutations or amino acid substitutions represented by symbols on the lines. Variations are relative to a reference sequence that in this case is a line without symbols. The center distribution describes an alignment of consensus sequences or sequences currently available in data banks. Each line is in reality an average of many different sequences with mutations additional to those included.

We examined for the same NS3 and NS5A amino acid stretches whether the amino acid subset that ranked as conserved in patients’ HCV quasispecies was also conserved in the LANL alignment, or whether the two conservation scores were unrelated. The results (Figure 5) show that the great majority of amino acids that were conserved among the different mutant spectra fell into the 90–100% LANL conservation group, and none below the 80–90% conservation group. This new calculation underlines the advantage of expanding the number of viral sequences that enter data banks to include mutant spectrum data [16,44]. Such an expansion will provide a more realistic assessment of residue conservation than current alignments of consensus sequences (Figure 6). Expanded amino acid sequence alignments, with a more stringent conservation criterion due to inclusion of mutant spectrum information should contribute more realistic data sets to attempt the design of universal antiviral ligands and vaccines. Although long-term avoidance of HCV escape mutants seems unlikely and contingent upon the extent of virus circulation [45,46], their selection may be delayed with designs based on super-conserved residues (Figure 6).

## 5. Antiviral Resistance Mechanisms during Hepatitis C Virus Infections

The efficacy of direct-acting antiviral (DAA)-based therapies to treat HCV infections is an unprecedented milestone in the hepatology field, and a remarkable success of clinical virology. Only 2% to 5% of DAA-treated patients do not respond to current treatments, often with the concomitant selection of virus with resistance-associated substitutions (RAS). RAS can be found not only in virus from DAA-treated patients but also in viruses from patients who had not been exposed to DAAs ([47,48,49], among other studies). Our recent research on HCV drug resistance both in cell culture and in HCV-infected patients has revealed three mechanisms of antiviral resistance.

The first mechanism entails the selection of bona fide RAS (list updated in the EASL guidelines [50]), which is an inhibitor-escape mechanism that has been historically documented for many important pathogenic RNA viruses, notably picornaviruses, HIV-1, and influenza virus [51]. Real-life data of HCV RAS from viruses circulating in infected patient cohorts who failed antiviral therapies are continuously being monitored [52,53,54]. We analyzed by Illumina UDS HCV RNA a Spanish cohort of 220 infected patients who failed DAA therapies [13]. To characterize the mutant spectra of these isolates, we designed subtype-specific oligonucleotide primers, performed the controls to define a cut-off mutant frequency for reliable detection of minority mutations, and implemented bioinformatics pipelines to derive a list of RAS and their frequencies within mutant spectra [11]. Both individual RAS and RAS combinations were viral subtype- and treatment-specific [13], a picture that agrees with that reached with other patient cohorts [52,53,54]. Moreover, contrary to some proposals that denied their clinical impact, RAS present at low frequencies within the viral quasispecies may contribute to treatment failures. Supporting observations are that 19% of the RAS described in our 220 patient cohort were found at frequencies below 20% in the viral quasispecies [13], and that, in two patients who did not respond to ledipasvir plus sofosbuvir therapy, the RASs imposed in the post-treatment sample were present at frequencies below 10% in the basal sample [55]. Therefore, UDS analysis of the resident virus is advisable prior to implementation of a new treatment, to minimize selection of RAS that are not detected in the consensus sequence.

A second type of antiviral resistance was recently discovered by our group, in the same cohort of 220 HCV-infected patients who failed DAA therapies [15]. It was reflected in a number of highly represented amino acid substitutions (HRSs), identified in NS5A and NS5B, not necessarily in the presence of RAS. HRSs were present in basal and post-treatment HCV samples and, contrary to RAS, they were not associated with escape to a specific treatment, but to resistance to multiple DAA treatments. In addition, HRSs appear to be subtype-specific as was previously defined for RAS mutations [13]. The HRS subset consisted of more conservative and tolerated amino acid replacements than the RAS subset. Although the occurrence of some HRSs has been described in other patient cohorts [56,57,58,59,60], additional studies are required to establish if they can have a predictive value of treatment failures in the absence of RAS.

A third type of antiviral resistance in HCV has been associated with the increase in viral fitness that results from passage of the virus in the constant cellular environment provided by Huh-7.5 hepatoma cells [12,18,19,43]. This mechanism does not depend on the selection of specific RAS or HRS, and it might be driven by constellations of fitness-enhancing mutations. It was discovered by serendipity when we observed that HCV p100, but not its parental HCV p0, displayed partial resistance to IFN-α despite not having been exposed to this cytokine [17]. Resistance was also manifested against several inhibitors such as telaprevir, daclatasvir, ribavirin, cyclosporine A, and sofosbuvir whose antiviral activity is exerted through different mechanisms of action [18,43]. Prior to our evidence of fitness-dependent drug resistance in HCV, we showed that high viral fitness conferred foot-and-mouth disease virus and human immunodeficiency virus type 1 resistance to lethal mutagenesis [61,62,63], but a general drug resistance was not demonstrated. In a study with HCV-infected patients subjected to daclatasvir monotherapy, it was suggested that viral fitness, rather than the presence of daclatasvir-resistance mutation, determined dominance of some HCV variants following therapy [64]. In our study on cell culture, the following analyses and control experiments excluded the participation of RAS in treatment failure: (a) UDS did not reveal substitutions catalogued as conferring resistance to the inhibitors used; (b) infections over a 1000-fold range of MOI did not alter the resistance profile, suggesting that resistance was not due to specific mutations present in genomes that would have been lost upon virus dilution; and (c) consistent with the previous dilution control, biological clones displayed similar levels of drug resistance than the parental high fitness HCV population from which they were isolated [18]. The absence of specific mutations is compatible with fitness increase of HCV in Huh-7.5 cells being achieved through multiple mutational pathways at many genomic sites [17].

There is the possibility that the presence of HRSs in patients who fail therapy is connected with the fitness-dependent mechanism of antiviral resistance recognized in cell culture, if the number of fitness-enhancing mutations in vivo is more restricted than in Huh-7.5 cells. However, HCV fitness comparisons in vivo are challenging and indirect at best, so that this possibility remains unproven [15]. These recent studies with HCV suggest that viruses may use mechanisms of antiviral resistance alternative to the profusely documented RAS selection.

## 6. A Unifying Disequilibrium Model for Expanded Adaptive Potential

The three major observations summarized here that comprise the occurrence of mutational waves, the discordances in residue conservation between quasispecies and consensus sequences stored in data banks, and RAS-independent drug-resistance mechanisms, may have a common underlying origin. We suggest that such a cause is a permanent population disequilibrium that pushes HCV to explore new regions of sequence space even when external selective constraints are absent. According to theoretical studies on quasispecies dynamics, in molecular evolution, the assumption of a constant environment is an idealized condition that cannot be realized since the individuals of a population of molecules contribute to the environment of the population [65]. In the case of HCV replication in cell culture, an internal effect of the mutant spectrum within individual infected cells or replicative organelles may be triggered by the random occurrence of mutations that renders the replicative context unique at each successive time point. This confers the mutant spectrum with an active role in the evolutionary process. The disequilibrium is maintained by mutational input, and impels the mutant spectrum to travel in sequence space, even without the need to respond to a selective constraint. Our interpretation is that the persistent disequilibrium gives rise to mutational waves, blurs the concept of conservation as deduced from current data banks, and facilitates finding alternative viral phenotypes—for example, escape pathways. The most direct response to an antiviral agent in patients is to select for RAS when genetic and phenotypic barriers allow. However, when the availability of RAS is not immediate, or its occurrence entails a significant fitness cost, alternative mechanisms become an option when disequilibrium allows for reaching the adept regions of sequence space.

If the mutant spectrum is part of the environment, then the impact of external perturbations in promoting mutant spectrum variation vanishes, since the perturbations are internally permanent. In our serial passages of HCV in cell culture, the presence of favipiravir or ribavirin (external perturbations) resulted in the enhancement—but was not a condition for—mutational waves. The viral population faces an ever changing environment that pushes the mutant spectrum towards increasing broadness, even if the viral population size remains constant. We have referred to this intrinsic property of an evolving viral quasispecies as “broadly diversifying selection”, built upon a progressive increase in the number of variant genomes that approach dominance in the population [42]. It is important to note that this enlargement of mutant repertoires was not restricted to neutral areas of sequence space since it affected phenotypic traits, not only of the entire population but also of clones within a population, as evidenced by differences in drug resistance levels [14]. A difference with classic positive selection is that it does not bring about dominance of genomes apt to confront a specific constraint, but it prepares the virus to respond to a range of environmental alterations still to come. The events can be viewed as a weakening of negative selection, rendering them closer to an extension of diversifying selection than to stabilizing selection as defined in general genetics [42].

Continuing work on HCV population dynamics with improved whole HCV genome quasispecies analyses are required to endorse our model and its implications. Since broadly diversifying selection has found support also with foot-and-mouth disease virus dynamics upon long-term passage in BHK-21 cells [42], it is tempting to propose that the model may be valid for other highly variable viral pathogens.

## 7. Concluding Remarks: Remembering John Holland in COVID-19 Times

The intrinsic population disequilibrium suggested by HCV mutant spectrum analyses accentuates even further the difficulties for viral disease prevention and control that we have previously emphasized [51,66,67]. Uncertainties for disease management due to individual variations that result in population heterogeneity may also apply to cells: in particular, to the diverse prokaryotic and eukaryotic microbial pathogens, as well as cancer cells, although in these cases affected by different genetic variation-genome size-population size parameters [51]. UDS and single cell analyses are forcing a rather annoying acceptance of a remarkable extent of individual variations in the viral and cellular worlds, prompting inquiries into their biological significance.

Descriptions of diversity—often considered unexpected and surprising—continue to this very day. Premonitory statements written by John Holland and his colleagues three decades ago have been dramatically confirmed during the COVID-19 pandemic, highlighted by the variety of disease symptoms associated with SARS-CoV-2 infection, and the target organs involved [68,69]. These sentences written in 1992 are, for instance, relevant at the present time, “There is an unspoken assumption among many physicians and scientists that *a particular RNA* virus will generally cause *a particular disease*. This assumption may be true in a very broad practical sense, but it is important to understand that it can never be true in a formal scientific sense. Because *a particular RNA virus* simply does not exist, *a particular virus disease* does not exist either. The science of infectious diseases is still in its infancy because we still understand so little of the fine details of host pathogen interactions” [70] (emphases as in the original text). Later in the same article, in connection with quasispecies and viral disease sequelae, the authors wrote, “Therefore, the acute effects and subtle chronic effects of infections will differ not only because we all vary genetically, physiologically and immunologically, but also because we all experience a different array of quasispecies challenges. These facts are easily overlooked by clinicians and scientists because disease syndromes are often grossly similar for each type of virus, and because it would appear to make no difference in a practical sense. However, for the person who develops Guillain–Barré syndrome following a common cold, or for the individual who remains healthy despite many years of HIV-1 infection, for example, it may make all the difference in the world”.

The perception of an infectious disease, its manifestations in different individuals, and the ways to define it, have been a matter of debate at various levels, even raising the issue of to what extent a disease exists as a specific entity [71]. In the deep sequencing era, in which individual virus and host differences become so obvious at the molecular level, the debate revives. The reflections on viral disease identity bring up a more general issue of the pursuit of unalterable definition of concepts in the face of the individual variations of the objects that construct the concepts. This problem and how viral quasispecies enter the debate are beyond the scope of the present article but will be presented elsewhere (J. Gomez et al. manuscript in preparation). We will need to refresh the concepts expressed by John Holland and colleagues for many years and pandemics to come.

## Figures and Tables

**Table 1:** Main observations on HCV population dynamics upon extended replication in human hepatoma Huh-7.5 cells.

Long-term replication in a non-coevolving cellular environment did not result in HCV population equilibrium. Changes in mutant frequencies (mutational waves) persisted despite increased adaptation to the cell culture environment [12]
High HCV fitness confers resistance to non-mutagenic inhibitors, and to the lethal mutagens favipiravir and ribavirin [19]
Short term mutational waves were also observed, suggesting that the system develops stochastic perturbations to enhance survival in case of unpredictable change [12,14]
Highly represented substitutions (HRS), different from resistance-associated substitutions (RAS), were identified in virus from patients who failed direct acting antiviral therapies [15]
A discrepancy between conserved residues in the Los Alamos database and the mutant spectra of infected patients was observed. This discrepancy could have a negative impact on the efficacy of universal vaccines and pan-genomic antiviral agents [16]

## Supplementary Materials

The following are available online at https://www.mdpi.com/article/10.3390/v13040616/s1, Table S1: List of amino acids that are strictly conserved in the HCV-infected patients.
