# Supplementary material for: Population Disequilibrium as Promoter of Adaptive Explorations in Hepatitis C Virus
Source: Viruses. 2021 Apr 3;13(4):616. doi: 10.3390/v13040616 (PMC8067247; doi:10.3390/v13040616)
Supplement: Supplementary file 1 [file viruses-13-00616-s001.pdf]

Table S1. List of amino acids that are strictly conserved in the HCV-infected patients[a].

Region	Number of conserved positions	Conserved positions[b]
**NS3**	40	32, 41, 44, 45, 53, 57, 58, 59, 60, 70, 73, 75, 77, 82, 85, 90, 97, 100, 106, 111, 118, 126, 129, 131, 135, 137, 138, 140, 141, 142, 145, 148, 149, 152, 157, 160, 161, 162, 164, 173
**NS5A**	24	29, 32, 33, 42, 45, 47, 51, 57, 65, 70, 76, 80, 82, 86, 89, 96, 106, 109, 118, 128, 131, 141, 142, 152

[a] The clinical history of the 220 patients cohort under study was described in Chen et al., Antiviral Research 174: 104694, 2020.

[b] The HCV genome residue numbering corresponds to the H77 genome (accession number #AF009606).
